# Quantitative Analysis Using Consecutive Time Window for Unobtrusive Atrial Fibrillation Detection Based on Ballistocardiogram Signal

**DOI:** 10.3390/s22155516

**Published:** 2022-07-24

**Authors:** Tianqing Cheng, Fangfang Jiang, Qing Li, Jitao Zeng, Biyong Zhang

**Affiliations:** 1 College of Medicine and Biological Information Engineering, Northeastern University, Shenyang 110819, China; 2171227@stu.neu.edu.cn (T.C.); 20195984@stu.neu.edu.cn (Q.L.); 20196008@stu.neu.edu.cn (J.Z.); 2 College of Medicine and Biological Information Engineering, Eindhoven University of Technology, 5600 MB Eindhoven, The Netherlands; biyong.zhang@slaaplekker.cn; 3 BOBO Technology, Hangzhou 310000, China

**Keywords:** recurrence plot, recurrence quantification analysis, continuous time windows, atrial fibrillation detection, ballistocardiogram signal

## Abstract

Atrial fibrillation (AF) is the most common clinically significant arrhythmia; therefore, AF detection is crucial. Here, we propose a novel feature extraction method to improve AF detection performance using a ballistocardiogram (BCG), which is a weak vibration signal on the body surface transmitted by the cardiogenic force. In this paper, continuous time windows (CTWs) are added to each BCG segment and recurrence quantification analysis (RQA) features are extracted from each time window. Then, the number of CTWs is discussed and the combined features from multiple time windows are ranked, which finally constitute the CTW–RQA features. As validation, the CTW–RQA features are extracted from 4000 BCG segments of 59 subjects, which are compared with classical time and time-frequency features and up-to-date energy features. The accuracy of the proposed feature is superior, and three types of features are fused to obtain the highest accuracy of 95.63%. To evaluate the importance of the proposed feature, the fusion features are ranked using a chi-square test. CTW–RQA features account for 60% of the first 10 fusion features and 65% of the first 17 fusion features. It follows that the proposed CTW–RQA features effectively supplement the existing BCG features for AF detection.

## 1. Introduction

Atrial fibrillation (AF) is a common type of arrhythmia, of which the incidence is increasing year by year [1,2]. There are currently 335 million individuals suffering from AF worldwide, with an overall prevalence rate of 2.9% [3,4]. It is particularly important to study the daily real-time and continuous monitoring and diagnosis of AF. At present, AF is widely detected using electrocardiograms (ECGs), the current gold standard for diagnosis [5,6,7,8]. However, ECG-based AF detection has been restricted by the need to attach electrodes on the body surface. Although wearable devices such as smartwatches provide a convenient way of collecting ECG data for daily monitoring, the principle of ECG detection is based on electrophysiology, which is unable to characterize the dynamic changes of the heart activity [9]. Therefore, unobtrusive methods based on cardiac dynamics have been brought into focus, including ballistocardiogram (BCG), seismocardiogram (SCG), and photoplethysmogram (PPG) methods [10,11,12].

Considering that the waveform components of PPG are less complex than those of SCG and BCG, SCG sensors need to be attached to the chest, so the AF detection method based on BCG is widely studied, which reflects the status of the larger cardiovascular system [13,14]. BCG is a noninvasive technique for recording the weak vibration signal on the surface of the body transmitted by the cardio-dynamic force [15]. Over the past decade, the development of BCG-based AF diagnosis through machine learning (ML) and deep learning (DL) has been increasingly investigated [16]. Bruser et al. extracted 17 time and time-frequency features of BCG signals from 10 patients, which were split into 856 nonoverlapping epochs with a length of 30 s each, and applied seven ML algorithms to classify AF, sinus rhythm (SR), and body movement [17]. Yu et al. recorded 12 AF patients’ data, and each BCG recording was split into nonoverlapping 30-s epochs, from which 12 power spectrum features were extracted. In addition, three ML classifiers were applied to distinguish AF and non-atrial-fibrillation (NAF) [18]. Wen et al. collected BCG signals from 37 subjects, which were split into 2915 1-min segments without overlap. Further, 16 energy features were extracted to classify AF and SR by means of five ML algorithms [19]. To substitute the feature extraction, the DL method has been applied to detect AF recently. In our previous study, the Bi-directional Long Short-Term Memory (Bi-LSTM) and phase space reconstruction (PSR) algorithm were employed to represent the 1-D morphology feature and 2-D rhythm feature of BCG signals, respectively, which were integrated by means of a CNN network to improve the robustness of AF detection [20]. In summary, the BCG-based AF detection method generally includes three main procedures: signal acquisition and preprocessing, feature extraction and selection, and classification and performance evaluation. Thereinto, the feature extraction is crucial, including the time feature, frequency feature, time-frequency feature, and non-linear feature.

The recurrence plot (RP) as a non-linear feature was first formally proposed by Eckmann et al. in 1987 [21], which maps a multi-dimensional nonlinear dynamic system by a two-dimensional graph. Recurrence quantification analysis (RQA) is an effective nonlinear tool, which is used to represent the non-stationary characteristics of signals and extract the nonlinear characteristic parameters of waveforms [22]. Currently, RP and RQA have been successfully applied in ECG-based arrhythmia detection. Sun et al. effectively predicted spontaneous termination of AF based on the structure and quantification of RP via ECG signals [23]. Mathunjwa et al. utilized ECG-based RP diagrams and convolutional neural networks (CNNs) to classify arrhythmias [24]. Chen et al. investigated cardiovascular activity changes under exposure to low-frequency noise for various noise intensities by using RP analysis of heart rate variability (HRV) estimation [25].

Considering that BCG has the same rhythm as ECG, we reconstruct the RP of the BCG signal and propose novel RQA features using continuous time windows (CTWs) to improve the accuracy of AF classification effectively in this study. In addition, we collect 4000 BCG segments from 59 subjects, which is more than in previous studies. As a comparison, the classical time and time-frequency features [17] and up-to-date energy features [19] are extracted using the BCG dataset of this study. From the contrast results, the AF classification accuracy of the proposed continuous time window RQA (CTW–RQA) features is superior to the accuracy of the other two existing features. Moreover, the CTW–RQA features are combined with the two types of existing features to constitute the fusion features, which achieve the optimal classification accuracy of 95.63%. To evaluate the importance of the proposed feature, the fusion features are ranked, and the CTW–RQA features account for 60% of the first 10 fusion features and 65% of the first 17 fusion features. It follows that the proposed CTW–RQA features effectively supplement the existing BCG features for AF detection.

The remainder of the paper is organized as follows. The Methods section elaborates the proposed approach including the signal acquisition and preprocessing, RP reconstruction, and CTW–RQA feature extraction and feature fusion. The experimental results are shown in the Results. In the Discussion, the experimental results are analyzed comprehensively. Finally, we summarize the drawbacks and future work in the Conclusions.

## 2. Methods

**Figure 1:**
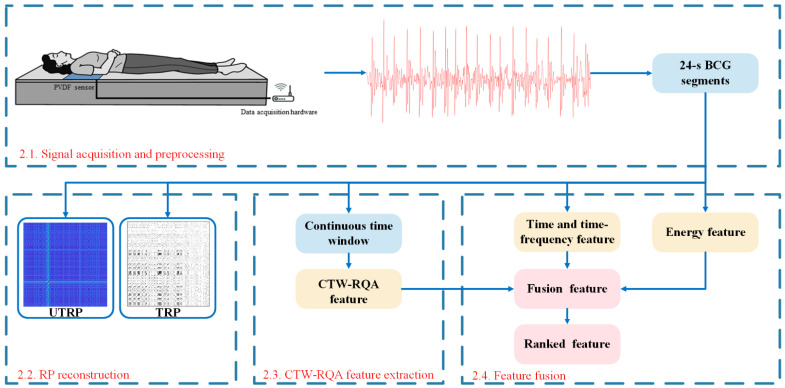
The process of the proposed method in this paper, where 2.1, 2.2, 2.3, and 2.4 represent the corresponding sections in the Methods.

The framework of the proposed method is shown in Figure 1.

### 2.1. Signal Acquisition and Preprocessing

BCG is a non-intrusive measurement of the vibration generated from the human heartbeat and arterial aortic blood circulation, which has the same rhythm as an ECG. In this study, BCG signals were recorded by means of the acquisition system that consisted of polyvinylidene fluoride (PVDF) and data acquisition hardware. The PVDF sensor (W × H: 30 cm × 60 cm, piezoelectric constant > 2 × 10^−11^ C/N) was placed on the top of a regular bed mattress and located underneath the subjects’ thorax with a sampling rate of 125 Hz (see 2.1 of Figure 1). During the recording process, the raw BCG waveform was amplified, and a Butterworth band-pass filter [17,26] was designed with a passband frequency of 0.7 Hz to 10 Hz to remove the high-frequency noise and low-frequency respiratory components and the motion artifacts, which was aimed at achieving a pure BCG signal. The ECG signal was collected by a CT-08S Holter Recorder at a sampling rate of 200 Hz. In order to address the problem of different sampling rates with BCG signals, the ECG signal was down-sampled to 125 Hz based on synchronized time stamps.

In total, 59 volunteers with paroxysmal AF, aged between 27 to 93 years, participated in this study; there were 34 males and 25 females. The BCG signal of each subject was recorded from 8 pm to 8 am in a lying position. Medical experts manually labeled the BCG signal as AF periods and NAF periods, taking synchronized ECG signals as the gold standard. AF periods and NAF periods were split to 24-s BCG segments, and 2000 AF segments and 2000 NAF segments were achieved as the BCG dataset. For AF classification, 80% of the BCG data were applied to train the classifiers, and the remaining 20% were recognized as independent testing data. In order to ensure the fairness of the experiments, all segments were collected from 59 subjects as evenly as possible. Additionally, the training and testing datasets were derived from different subjects to avoid overfitting.

### 2.2. RP Reconstruction

**Figure 2:**
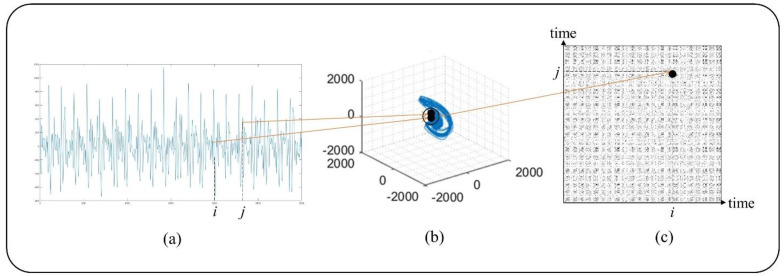
RP reconstruction schematic diagram: after the BCG signal is transformed into phase space trajectory, RP is obtained through RP reconstruction, where (**a**) is the waveform of the 24-s BCG segment, (**b**) is the phase space trajectory of BCG, and (**c**) is the RP of BCG.

Recurrence is a fundamental property of dynamic systems, which can be exploited to characterize the system’s behavior in phase space [27]. A powerful tool for their visualization and analysis, called RP, was introduced in the late 1980s by Eckmann, which reveals all the times when the phase space trajectory of the dynamic system visits roughly the same area in the phase space [21]. Cardiac activity is a dynamic system, so RP is used for nonlinear analysis of the BCG rhythm in this study. Figure 2 shows the BCG reconstruction process.

The basis of RP reconstruction is to reconstruct the phase space. Takens theorem states that a phase space can be reconstructed by the system component after selecting the appropriate delay time *τ* and embedding dimension *m* for the time series, which contains all of the information of the original time series [28].

Set the BCG time series {$u_{1},u_{2},\ldots ,u_{n}$}, and then the *m*−dimensional BCG reconstructed vector $x_{i}$ is defined as Equation (1).

(1) $$x_{i}=\left[u_{i},u_{i+\tau },\ldots ,u_{i+\left(m-1\right)\tau }\right]\left(i=1,2,\ldots ,n-\left(m-1\right)\tau \right)$$

where *m* is the embedding dimension and *τ* is the delay time, *i* = 1, 2, …; *n*−(*m*−1) *τ*. In this study, the mutual information method is used to determine the parameter *τ*, and the pseudo-neighborhood method is used to determine the parameter *m*, which have been discussed in literature [20].

As shown in Figure 2, the distance between the points i and j in the reconstructed phase space can be calculated with Equation (2).

(2) $$S_{ij}=∥x_{i}-x_{j}∥\left(i=1,\ldots ,n-\left(m-1\right)\tau ,j=1,\ldots ,n-\left(m-1\right)\tau ,\;x_{i}\in {\mathbb{R}}^{m}\right)$$

The RP is a collection of time pairs at the same position in the phase space trajectory in the two-dimensional time domain as shown in Equations (3) and (4).

(3) $$R_{i,j}^{m\cdot \varepsilon_{i}}=\Theta \left(\varepsilon_{i}-S_{ij}\right)=\;\Theta \left(z_{ij}\right)\left(i,j=1\ldots N\right)$$

(4) $$\Theta \left(z_{ij}\right)=\{\begin{array}{c} 0\;\;\;\;\;if\;z_{ij}>0\\ 1\;\;\;\;\;\mathrm{otherwise} \end{array}$$

where the $R_{i,j}^{m\cdot \varepsilon_{i}}$ is the recursion value, and $z_{ij}$ represents the difference between threshold $\varepsilon_{i}$ and $S_{ij}$.

**Figure 3:**
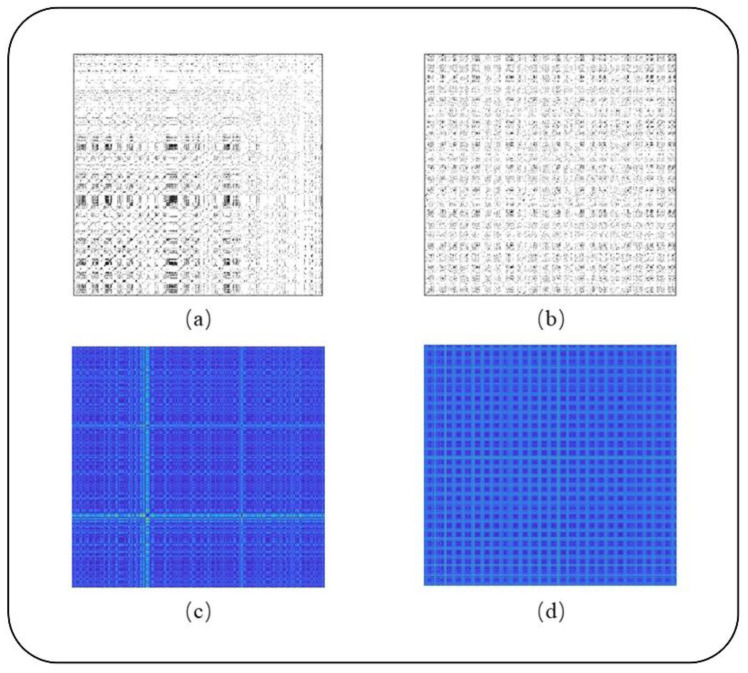
TRP and UTRP of AF and NAF. (**a**) is the TRP of AF, and (**b**) is the TRP of NAF; (**c**) is the UTRP of AF, and (**d**) is the UTRP of NAF.

Equation (4) shows that for the distance between the *m*-dimensional trajectory of time *j* and the *m*-dimensional trajectory of time *i*, a black point is placed at the coordinates (*i*, *j*), otherwise, a white point is placed. A key parameter in the analysis is the threshold $\varepsilon_{i}$, which is chosen as 10% of the maximum phase space diameter based on the theory of the RP [27,29,30,31]. The thresholded recurrence plots (TRPs) of AF and NAF are drawn in Figure 3a,b. In another case, without setting the threshold, the value of point (*i*, *j*) in the image matrix is calculated as the Euclidean distance between point *i* and point *j* in the phase space. The unthresholded recurrence plots (UTRPs) of AF and NAF are shown in Figure 3c,d.

### 2.3. CTW–RQA Feature Extraction

**Figure 4:**
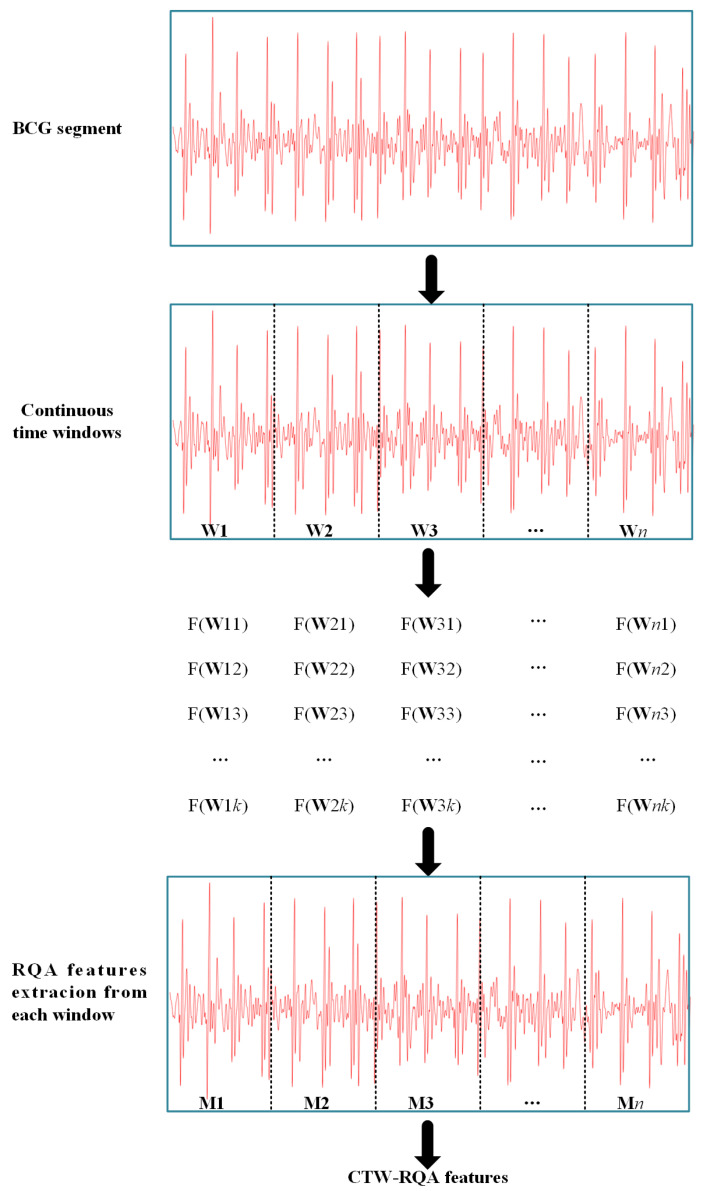
The process of CTW–RQA feature extraction.

RQA defines new measures of complexity by using geometrical structures of RP. According to the characteristic of phase space trajectories, recursive graphs contain typical small-scale structures such as single points, diagonals, vertical lines, and horizontal lines, which constitute the large-scale texture structures. RQA quantifies the small-scale and the large-scale texture structures [31]. In this paper, *n* consecutive time windows are designed to divide each BCG segment, which refine the region of interest for the original signal. Afterwards, *k* RQA features are extracted in each time window to constitude the CTW–RQA feature. The feature extraction process is shown in Figure 4.

In Figure 4, **W***i* are the time windows to be analyzed (*i* = 1, 2, …, *n*). **W***ij* refers to the *j*th RQA feature extracted from the *i*th time window (*j* = 1, 2, …, *k*), *k* is the number of RQA features. **M***i* represents the feature vector composed of *k* RQA features in the *i*th time window, and the CTW–RQA feature of each BCG segment is defined in Equation (5).

CTW–RQA = [**M**1,**M**2, …,**M***n*] (5)

In this study, *k* is selected as 13, and the 13 RQA features are shown in Table 1, which are denoted as RQA1, RQA2, …, RQA13.

In Table 1, *RR* is the percentage of recurrence points in an RP; *DET* is the percentage of recurrence points that form vertical lines, where the *P*(*l*) is the histogram of the lengths *l* of the diagonal lines; *LAM* is the percentage of recurrence points that form vertical lines, where *P*(*v*) is the histogram of the lengths *v* of the vertical lines; *RATIO* is the ratio between *DET* and *RR*; *L* is the expression of the average length of the diagonal lines; *TT* is the percentage of the average length of the vertical lines; $L_{max}$ is the expression of the length of the longest diagonal line; *V_max_* is the expression of the length of the longest vertical line; *DIV* is the reciprocal of $L_{max}$; ENTR is the percentage of the Shannon entropy of the probability distribution of the diagonal line lengths *P*(*l*); *TREND* is the percentage decrease of the *RP* towards its edges; *CLUST* is the percentage of the ratio of the number of closed triplets to the number of all triplets; $WV_{max}$ is the percentage of the length of the white vertical line.

### 2.4. Feature Fusion

In order to validate the characterization performance of the proposed features, 17 time and time-frequency features and 16 energy features are extracted in this section. And the three types of features are ranked, and finally the fusion features and the ranked features are derived to improve the accuracy of AF detection.

#### 2.4.1. Time and Time-Frequency Features and Energy Feature Extraction

In the literature [17], 6 time features and 11 time-frequency features were extracted from BCG signals, which were fed into 7 popular ML classifiers to detect AF. The definitions of the 17 time and time-frequency features are shown in Table 2, which are denoted as TTF1, TTF2, …, TTF17.

In Table 2, $pp_{10}\left(l\right)$ is defined as the peak-to-peak amplitude of ten segments that is split from 24-s BCG segments. S[f,t] represents the result of the energy spectral density calculation of data by defining a 5-s window, S(f) represents the result of the time mean of S[f,t], $f_{\mathrm{peak}\;}$ represents the distance between peaks of S[f,t], and $w_{\mathrm{peak}\;}\left[k\right]$ represents the distance from the *k*th peak to the average peak height.

In the literature [19], BCG signals were transformed into BCG energy signals in order to highlight the features of AF and NAF, and four new data sequences representing different characteristics of the BCG energy signals were generated. The mean value, variance, skewness, and kurtosis of the four data sequences were calculated, and 16 energy features were extracted for each BCG segment. Five ML algorithms were used to distinguish AF and NAF. The definitions of the 16 energy features are shown in Table 3, which are denoted as E1, E2, …, E16.

In Table 3, *P*(i) values are sample points that correspond to peaks, where *i* indicates the *i*th peak in the segment; *T*(i) comprises the coordinates of troughs; *DA*(*i*) denotes the relative difference of the peak amplitude; *RT*(i) stores the relative value of the trough amplitude; and *BP*(i) was defined to denote the number of burrs between *P*(i) and *P*(i+1).

#### 2.4.2. Feature Ranking and Selection

In order to evaluate the features proposed, four types of features are ranked using the Fisher Score, chi-square test, minimum redundancy–maximum relevance (MRMR) and SHapley Additive exPlanations (SHAP) algorithms.

The Fisher Score is defined as the ratio of inter-class variance to intra-class variance [32], which is calculated as Equations (6)–(8). It can be deduced that when the inter-class variance is larger and the intra-class variance is smaller, the Fisher Score is larger. Therefore, higher ranked features are more discriminatory theoretically.

(6) $$S_{B}^{\left(k\right)}=\overset{C}{\underset{i=1}{\sum }}\frac{n_{i}}{n}{\left(m_{i}^{\left(k\right)}-m^{\left(k\right)}\right)}^{2}$$

(7) $$S_{w}^{\left(k\right)}=\frac{1}{n}\overset{C}{\underset{i=1}{\sum }}\underset{x\in w_{i}}{\sum }{\left(x^{\left(k\right)}-m_{i}^{\left(k\right)}\right)}^{2}$$

(8) $$J_{\mathrm{fisher}\;}\left(k\right)=\frac{S_{B}^{\left(k\right)}}{S_{w}^{\left(k\right)}}$$

where $x^{\left(k\right)}$ represents the value of sample *x* for the *k*th feature, $m_{i}^{\left(k\right)}$ represents the mean of the values of class *i* samples for the kth feature, and $m^{\left(k\right)}$ represents the mean of the values of all categories of samples for the kth feature. $S_{B}^{\left(k\right)}$ is defined as the inter-class variance of the *k*th feature in the data set; $S_{w}^{\left(k\right)}$ is defined the intra-class variance of the *k*th feature in the data set; and $J_{\mathrm{fisher}\;}\left(k\right)$ defines the Fisher Score of the *k*th feature in the data set.

The chi-square test is a common hypothesis testing method based on the χ^2^ distribution of the test statistic. Its null hypothesis H0 is that the observed frequency does not differ from the expected frequency. The basic idea of this test is as follows: assume that H0 is established; then, calculate the χ^2^ value based on this premise, which represents the degree of deviation between the observed value and the theoretical value. According to the χ^2^ distribution and degrees of freedom, the probability p of obtaining the current statistic and more extreme cases can be determined under the condition that the H0 hypothesis holds. The chi-square test checks whether each feature is independent of the label. A small *p*-value for the test statistic indicates that the corresponding feature is dependent on the label, proving that the feature is important [33]. To amplify the difference between features, importance scores are proposed, as shown in Equation (9).

(9) Importance scores=−lg(p)

The MRMR algorithm finds an optimal set of features that are maximally different from each other and that can effectively represent the label variable [34]. The algorithm calculates the mutual information between features and the mutual information between features and labels to quantify redundancy and correlation. The features are ranked according to the criteria of minimizing the redundancy of the feature set and maximizing the correlation between the feature set and the label variable.

The SHAP algorithm can interpret each feature’s importance to predictions [35]. The SHAP value has been used to guide feature selection [36], which explains the deviation of the prediction for the query sample from the average prediction. In this algorithm, the model needs to be retrained on all feature subsets S ⊆ F, where F represents all features. It assigns an importance value to each feature that represents the effect on the model prediction when including that feature. To compute this effect, a model $f_{S\cup^{\;}\left\{i\right\}}$ is trained with that feature present, and another model $f_{S}$ is trained with the feature withheld. Then, a comparison is conducted between the two prediction models with respect to the current input $f_{S\cup^{\;}\left\{i\right\}}\left(x_{S\cup^{\;}\left\{i\right\}}\right)-f_{S}\left(x_{S}\right)$ to calculate the SHAP values, where $x_{S}$ represents the input features in the value set S [35]. In this study, each BCG segment is used as $x_{S}$, and the average of the absolute values of the resulting SHAP values is used to draw a SHAP summary plot and is applied to feature ranking.

#### 2.4.3. Fusion Feature Extraction

**Figure 5:**
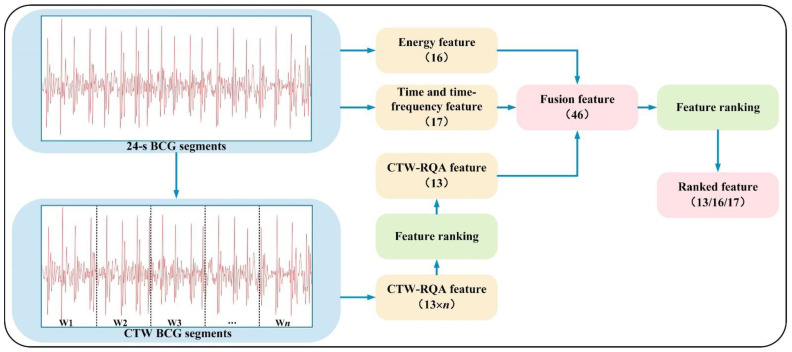
The extraction process of the proposed fusion features and ranked features.

Based on the ranking results of the CTW–RQA feature, the first 13 features are selected, which is consistent with the dimensionality of RQA features without CTW. Afterwards, 17 time and time-frequency features, 16 energy features, and 13 ranked CTW–RQA features are combined as 46 fusion features, which provide abundant characterization information. However, there must be irrelevant or redundant information in the fused features. Therefore, 46 fusion features are ranked holistically, and the first 13, 16, and 17 features are selected, which are denoted as the Top 13, Top 16, and Top 17 ranked features. The selected dimensions are consistent with the time and time-frequency features, energy features, and ranked CTW–RQA features. The purpose is to compare the classification performance of ranked features with each type of independent feature in the same dimension. Figure 5 demonstrates the extraction process of the proposed fusion features and ranked features.

## 3. Results

### 3.1. AF Detection Based on the RP Diagram

TRP and UTRP are reconstructed from 4000 BCG segments, which are fed into the designed CNN directly. The network mainly consists of eight convolution layers, four pooling layers, four dropout layers, one flatten layer, and one full connection layer and outputs the result of dichotomy, which is successfully performed for AF detection from the BCG signal [20]. The accuracy (ACC), sensitivity (SEN), precision (PRE), and specificity (SPE) are calculated to estimate the AF classification performance, which is shown in Table 4.

From Table 4, the classification performance of UTRP is superior to TRP. However, the classification accuracy of UTRP and TRP is less than 80%, which cannot keep up with the demand of AF screening in routine life.

### 3.2. AF Detection Based on CTW–RQA Features

Based on Table 1, 13 RQA features are extracted from each 24-s BCG segment, and five ML classifiers are used for training and testing, including K-Nearest Neighbors (KNN), Naive Bayes (NB), Ensemble Learning (ENS), Random Forest (RF), and a Decision Tree (DT) [19,37,38]. The AF detection performance is shown in Table A1 of the Appendix A. As a comparison, CTW–RQA features based on three different time window lengths are extracted, and *n* is set to 6, 3, and 2. The same ML classifiers are used to detect AF, and the performance is shown in Table A2, Table A3 and Table A4 of the Appendix A. From Table A1, Table A2, Table A3 and Table A4, RF is the optimal classifier, so Table 5 illustrates the AF detection performance based on RQA features and CTW–RQA features by means of RF.

As shown in Table 5, the accuracy of the RQA features and CTW–RQA features is higher than that of the CNN based on UTRP in Table 4. When *n* is selected as 3, the CTW–RQA features obtain the highest classification accuracy of 89.38%, which is superior to the RQA features without CTW.

### 3.3. AF Detection Based on Time and Time-Frequency Features and Energy Features

Based on Table 2, 17 time and time-frequency features (6 time domains and 11 time-frequency domains) are extracted from the 4000 BCG segments in this study. In addition, the 4000 BCG segments are transformed into BCG energy signals to extract 16 energy features based on Table 3. The same five ML algorithms are employed to classify AF and NAF. The classification performance is shown in Table A5 and Table A6 of the Appendix A.

From Table A5 and Table A6, RF achieves the optimal classification performance. Therefore, RF is applied to evaluate two types of features with CTW. As a comparison, *n* is also selected as 6, 3, and 2. The AF detection performance is shown in Table 6 and Table 7.

From Table 6 and Table 7, the time and time-frequency features and energy features without CTW exhibit a superior classification performance and are suitable for feature fusion.

### 3.4. AF Detection Based on Fusion Features

From Table 5, *n* is selected as 3. According to Method Section 2.4.2, the Fisher Score, chi-square test, MRMR, and SHAP algorithms are used for feature ranking. The calculated results of 13 RQA features based on four feature ranking algorithms are shown in Table 8.

According to Figure 5, the 39 CTW–RQA features are reduced to 13 CTW–RQA features based on Table 8. Then, 46 fusion features, composed of 13 CTW–RQA features, 17 time and time-frequency features, and 16 energy features, are fed into five ML classifiers to detect AF. The results are shown in Table 9.

As shown in Table 9, RF achieves the optimal classification accuracy, which is 95.63%.

### 3.5. AF Detection Based on Ranked Features

**Figure 6:**
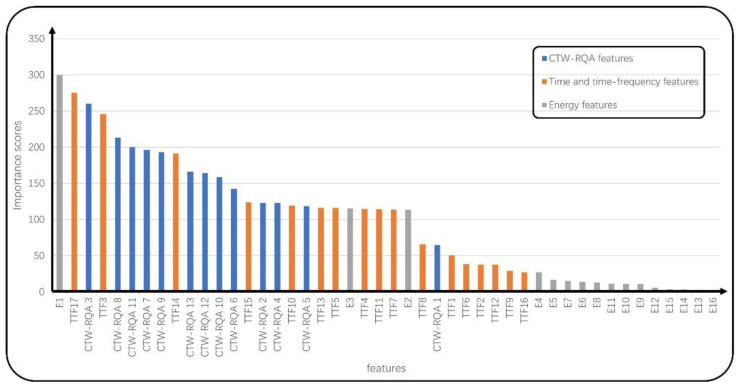
The ranking results of fusion features based on the chi-square test.

**Figure A1:**
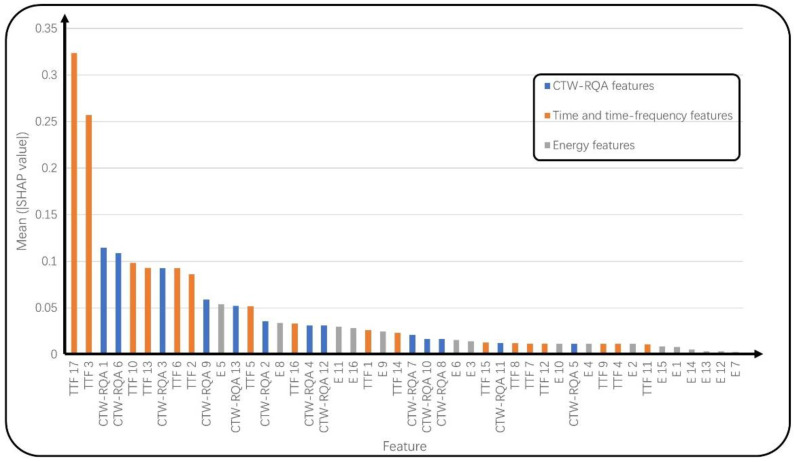
The ranking results of fusion features based on the SHAP summary plot.

From Table 9, the classification performance of the fusion features has been obviously improved. However, excessive feature dimensionality may affect the classification efficiency, so 46 fusion features are ranked by the same four feature ranking algorithms. Figure 6 shows the ranking results of the fusion features by the chi-square test method, and Figure A1 in the Appendix A shows the ranking results of the fusion features by the SHAP method.

According to Method Section 2.4.3, the Top 13, Top 16, and Top 17 ranked features are selected based on the ranking results of fusion features, which are fed into the optimal RF classifier. The AF detection accuracy based on the ranked features is shown in Table 10.

From Table 10, the ranked features selected by the chi-square test have the highest classification accuracy. The Top 17 ranked features have a comparable classification performance with the 46 fusion features, which validates the effect of feature selection.

## 4. Discussion

In this study, CTW–RQA features are proposed to improve the accuracy of AF detection based on BCG signals. The main contributions of our work are the following: (1) RP is first applied to detect AF by means of BCG signals. TRP and UTRP are fed into the designed CNN to classify AF and NAF. (2) The CTW–RQA features are proposed for the first time to quantify the AF rhythm characteristic, which are compared with the 17 classical time and time-frequency features and 16 up-to-date energy features using five ML classifiers. The AF classification accuracy of the proposed feature is superior to the other existing features using the BCG dataset of this study. (3) The CTW–RQA features are combined with the two types of existing features to constitute the fusion features. Then, the fusion features are sorted and selected to constitute the ranked features. The 46 fusion features achieve the optimal classification accuracy of 95.63%, and the 17 ranked features result in a decrease of only 0.25%. It follows that the feature ranking and selection are necessary, and the proposed CTW–RQA features effectively supplement the existing BCG features for AF detection. Furthermore, the reasons underlying the experimental results are analyzed in detail.

### 4.1. Effects of RP and RQA

From Table 4, the AF detection performance based on TRP and UTRP is compared by means of a CNN, and the results show that UTRP has superior classification accuracy, which is 73.25%. This may be because UTRP contains substantially more information from the signal that generated it than TRP [39]. However, the classification accuracy of both TRP and UTRP could not satisfy the requirement of AF screening. This result may be explained as follows: RP is a sparse image, and non-contiguous areas contain key features, which are difficult to extract by the convolution kernel of the CNN [40]. Therefore, RQA is utilized to emphasize textural features of the RP graph more concretely. From Table 5, 13 RQA features are extracted to input into 5 ML classifiers, and the RF classifier has the optimal performance with an accuracy of 83.50%, which is superior to TRP and UTRP. It follows that RQA features characterize the abnormal rhythm information of BCG signals effectively, which improves the accuracy of AF diagnosis. Therefore, RQA features with the ML classifier are taken as the basis for subsequent feature modifications.

### 4.2. Effect of the Proposed CTW–RQA Features

In Table 5, the AF classification accuracy of the CTW–RQA features with three types of time windows (*n* = 6, 3, 2) is compared to the accuracy of the RQA features. The CTW–RQA features with 12-s (*n* = 2) and 8-s (*n* = 3) time windows exhibit a higher AF detection accuracy than the RQA features without CTW. However, the CTW–RQA features with a 4-s (*n* = 6) time window exhibit worse performance. It follows that longer time windows are insensitive to small rhythm changes; conversely, shorter time windows may miss occasional rhythm abnormalities. Therefore, the CTW–RQA feature with 8-s time windows (*n* = 3) achieves the best classification performance.

Similarly, from Table 6 and Table 7, the time and time-frequency features and energy features without CTW show superior classification performance. This is since the time features are extracted to quantify the amplitude, location information of wave peaks, and inter-peaks in the BCG waveform, so time window addition may miss the effective information. Additionally, limited by the uncertainty principle [41], it is understood that a shorter CTW used to capture the signal segment leads to a poor resolution to represent the signal in the frequency domain, which in turn negatively affects the performance of AF detection. For the energy features, the BCG segments need to be transformed into BCG energy signals, which describe the change in energy distribution. Therefore, longer segmentation lengths are suitable to emphasize the distribution of energy. In summary, CTW is not added to the time and time-frequency features and energy features during feature fusion.

Compared to the time and time-frequency features and energy features without CTW, the proposed CTW–RQA features exhibit the optimal AF classification performance based on Table 5, Table 6 and Table 7. This is likely because the two existing features are more dependent on the variability of the waveform, whereas the data used in this study are derived from a larger number of subjects. Therefore, the proposed CTW–RQA features are more suitable for daily AF screening.

### 4.3. Effect of Fusion Features and Ranked Features

From Table 9, fusion features exhibit an optimal classification accuracy of 95.63%, which is improved significantly. This is because more dimensions and more types of features provide more information in general. Nevertheless, there is redundant information in the fusion features [42]. Therefore, feature ranking is applied to reduce the dimensions of features and to improve the efficiency of the model.

From Table 10, the features selected by the chi-square test achieve the highest accuracy. This is because this method intuitively quantifies the degree of deviation between features and labels [33] and accurately finds the features strongly correlated with the labels, which is suitable for feature selection in large data sets. Based on the results of the chi-square test, the Top 13, Top 16, and Top 17 ranked features with AF detection accuracy are 95.25%, 95.25%, and 95.38%, respectively, which are comparable to the 46 fusion features. Additionally, with the same feature dimension, the classification accuracy of ranked features is higher than 13 CTW–RQA features, 16 energy features, and 17 time and time-frequency features. The Top 17 ranked features reduce the feature dimensions to improve the efficiency of the algorithm, while the classification result is only 0.25% lower than the 46 fusion features. It reflects the superiority of the ranked features, and the feature selection is necessary and effective.

According to the ranking results based on the chi-square test in Figure 6, there are 6 CTW–RQA features from the first 10 features, and CTW–RQA features account for 65% of the first 17 ranked features, verifying the characterization ability of the CTW–RQA features. Moreover, the proposed features supplement the existing BCG features and effectively improve the performance of AF detection.

### 4.4. Comparison with Existing Methods

For AF detection based on BCG signals, Bruser et al. extracted the time and time-frequency features from 10 subjects, and the best classifier achieved an accuracy of 96% from 856 BCG segments [17]. Yu et al. extracted the time-frequency features from 12 subjects, and the optimized classifier achieved an accuracy of 94.4% from 7816 BCG segments [18]. Wen et al. extracted the energy features from 37 subjects, and the best classifier achieved an accuracy of 94.5% from 2915 BCG segments [19]. In this study, 4000 BCG segments from 59 subjects were collected, which is more diverse. The more diverse range of subjects enabled the proposed method to be more generalizable, which is more relevant to the application environment of AF screening. Additionally, the AF detection accuracy of proposed features is superior to the accuracy of existing features based on the BCG dataset in this study. The optimal classifier achieves an accuracy of 95.63%, which could satisfy the demand of AF diagnosing in routine life. In summary, the proposed features present an advantage in the field of AF detection based on more diverse BCG datasets, which effectively supplement the existing BCG features.

Compared to existing wearable AF detection devices, the superiority of BCG-based AF detection is as follows. Current technologies employed in wearables and evaluated for AF detection are most often based on single-lead ECG or PPG. For ECG-based AF detection, wearable devices include smartwatches/bands [43,44], smartphones [45,46,47], skin patch recorders [45], hand-held devices [46,47], and smart clothing [48]. These monitoring devices are convenient, but they are based on cardiac electrophysiology and lack the analysis of cardio-dynamic changes. For PPG-based AF detection, wearable devices include smartwatches/bands [49,50,51], smartphones [52,53], and cameras [54]. These monitoring devices can provide cardio-dynamic information, but the accuracy is currently affected by motion artefacts, measurement location, skin conditions, ectopic beats, peripheral vascular disease, poor skin contact, and limited battery life [44,55]. In contrast, BCG-based AF detection devices are usually cushions or mattresses [16], which keep individuals unaware of the test and reflect the status of the larger cardiovascular system [13,14].

However, the limitation of BCG-based AF detection is the presence of motion artefacts, thus stationary conditions are always desired. In state-of-the-art research, the BCG dataset is generally collected during sleep, where high signal-to-noise ratio periods cover most of the acquired data. Compared with the techniques using wearable devices, BCG-based AF detection does not introduce an addition burden. For example, some devices demand individuals to place their fingers on the electrodes of a smartphone [46,47]. Moreover, the AF prevalence increases with age, especially for those aged > 65 years, whereas only 4.6% of smartwatch users in the United States are aged > 65 years [56]. Therefore, BCG-based AF detection devices are more suitable for the elderly or bedridden patients.

## 5. Conclusions

In this study, we reconstruct BCG signals with the RP method and propose CTW–RQA features to optimize the AF classification performance. The experimental results prove that the proposed features are feasible for AF detection using BCG signals. In the future, we will expand the data volume of BCG signals, including multiple postures and more subjects, to verify the universality of the algorithm. Additionally, the data length for each subject will be increased for personalized analysis for specific subjects. For feature selection, more feature ranking methods, such as the SHAP dependence plot, could be applied to evaluate the importance of the BCG features. The CTW–RQA features and feature selection method in this paper could be extended for the diagnosis of other arrhythmia diseases by means of BCG signals, and the influence of respiratory movement could be considered.

## Figures and Tables

**Table 1:** The definition of 13 RQA features.

Symbols	Measure	Definition
RQA 1	Recurrence rate (*RR*)	$RR=\frac{1}{N^{2}}{\mathrm{sum}}_{i,j=1}^{N}R_{i,j}$
RQA 2	Determinism (*DET*)	$DET=\frac{\sum_{l-l_{min}}^{N}lP(l)}{\sum_{l=1}^{N}lP(l)}$
RQA 3	Laminarity (*LAM*)	$LAM=\frac{\sum_{v-v_{min}}^{N}vP(v)}{\sum_{v=1}^{N}vP(v)}$
RQA 4	Ratio (*RATIO*)	$RATIO=N^{2}\frac{\sum_{l-l_{min}}^{N}lP(l)}{{\left(\sum_{l-1}^{N}lP(l)\right)}^{2}}$
RQA 5	Averaged diagonal line length (*L*)	$L=\frac{\sum_{l-l_{min}}^{N}lP(l)}{\sum_{l-l_{min}}^{N}P(l)}$
RQA 6	Trapping time (*TT*)	$TT=\frac{\sum_{v-v_{min}}^{N}vP(v)}{\sum_{v=v_{min}}^{N}P(v)}$
RQA 7	Longest diagonal line ($L_{max}$)	$L_{max}=max\left(\left\{l_{i};i=1,\ldots ,N_{l}\right\}\right)$
RQA 8	Longest vertical line ($V_{max}$)	$V_{max}=max\left(\left\{v_{i};i=1,\ldots ,N_{v}\right\}\right)$
RQA 9	Divergence (*DIV*)	$DIV=\frac{1}{L_{max}}$
RQA 10	Entropy (*ENTR*)	$ENTR=-\overset{N}{\underset{l-l_{min}}{\sum }}p(l)\ln p(l)$
RQA 11	Trend (TREND)	$TREND=\frac{\sum_{i=1}^{N}(i-\tilde{N}/2)\left(RR_{i}-\langle RR_{i}\rangle \right)}{\sum_{i=1}^{N}{\left(i-\tilde{N}/2\right)}^{2}}$
RQA 12	Clustering coefficient (*CLUST*)	$CLUST=\frac{\mathrm{CTN}}{\mathrm{TN}}$
RQA 13	Longest white vertical line ($WV_{max}$)	$WV_{max}=max\left(\left\{wv_{i};i=1,\ldots ,N_{v}\right\}\right)$

**Table 2:** The definition of 17 time and time-frequency features.

Symbols	Measure	Definition
TTF1	Standard Deviation	$std\left(x\left[n\right]\right)=\sqrt{\frac{N}{N-1}m_{2}\left(x\left[n\right]\right)}$
TTF2	Skewness	$\mathrm{skewness}\;\left(x\left[n\right]\right)=\frac{m_{3}\left(x\left[n\right]\right)}{m_{2}{\left(x\left[n\right]\right)}^{3/2}}$
TTF3	Kurtosis	$kurtosis\left(x\left[n\right]\right)=\frac{m_{4}\left(x\left[n\right]\right)}{m_{2}{\left(x\left[n\right]\right)}^{2}}$
TTF4	Range	pp(x[n])=max(x[n])−min(x[n])
TTF5	$\mathrm{Ratio}\;\mathrm{of}\;pp_{10}\left(l\right)$ to the Mean	$\max\left(pp_{10}\left(l\right)/mean\left(pp_{10}\left(l\right)\right)\right)$
TTF6	$\mathrm{Standard}\;\mathrm{Deviation}\;\mathrm{of}\;pp_{10}\left(l\right)$	$\mathrm{std}\left(pp_{10}\left(l\right)\right)$
TTF7	$\mathrm{The}\;\mathrm{Standard}\;\mathrm{Deviation}\;\mathrm{of}\;\bar{S}\left[f\right]$	$\mathrm{std}\;\left(\bar{S}\left[f\right]\right)$
TTF8	$\mathrm{Skewness}\;\mathrm{of}\;\bar{S}\left[f\right]$	$\mathrm{skewness}\;\left(\bar{S}\left[f\right]\right)$
TTF9	$\mathrm{Kurtosis}\;\mathrm{of}\;\bar{S}\left[f\right]$	$\mathrm{kurtosis}\;\left(\bar{S}\left[f\right]\right)$
TTF10	$\mathrm{Standard}\;\mathrm{Deviation}\;\mathrm{of}\;f_{\mathrm{peak}\;}$	$std\left(\Delta f_{\mathrm{peak}\;}\left[k\right]\right)$
TTF11	$\mathrm{Skewness}\;\mathrm{of}\;f_{\mathrm{peak}\;}$	$\mathrm{skewness}\;\left(\Delta f_{\mathrm{peak}\;}\left[k\right]\right)$
TTF12	$\mathrm{Kurtosis}\;\mathrm{of}\;f_{\mathrm{peak}\;}$	$\;\mathrm{kurtosis}\;\left(\Delta f_{\mathrm{peak}\;}\left[k\right]\right)$
TTF13	Standard Deviation of S[f,k]	$std\left(std_{t}\left(S\left[f,k\right]\right)\right)$
TTF14	$\mathrm{Average}\;\mathrm{of}\;w_{\mathrm{peak}\;}$	$\mathrm{mean}\left(w_{\mathrm{peak}\;}\left[k\right]\right)$
TTF15	$\mathrm{Standard}\;\mathrm{Deviation}\;\mathrm{of}\;w_{\mathrm{peak}\;}$	$\mathrm{std}\left(w_{\mathrm{peak}\;}\left[k\right]\right)$
TTF16	Harmonic Drama Frequency	$\max_{fd}\overset{\left[\frac{F}{fb}\right]}{\underset{k=1}{\sum }}\log_{10}(\frac{\bar{S}m_{4}\left(kf_{b}\right)}{\bar{S}\left[\left(k+\frac{1}{2}\right)f_{b}\right]})$
TTF17	Kurtosis of Continuous Time Energy Spectral Density	$\overset{T-1}{\underset{t=1}{\sum }}\mathrm{kurtosis}\;(\mathrm{xcorr}(S[f,t],S[f,t+1]))$

**Table 3:** The definitions of the 16 energy features.

Symbols	Definition
E1	Mean (PI(i))
E2	Variance (PI(i))
E3	Skewness (PI(i))
E4	Kurtosis (PI(i))
E5	Mean (DA(i))
E6	Variance (DA(i))
E7	Skewness (DA(i))
E8	Kurtosis (DA(i))
E9	Mean (RT(i))
E10	Variance (RT(i))
E11	Skewness (RT(i))
E12	Kurtosis (RT(i))
E13	Mean (BP(i))
E14	Variance (BP(i))
E15	Skewness (BP(i))
E16	Kurtosis (BP(i))

**Table 4:** AF detection performance based on RP by means of the CNN.

Method	ACC	PRE	SEN	SPE
TRP	56.13	73.50	38.75	59.38
**UTRP**	**73.25**	**78.75**	**67.75**	**76.12**

**Table 5:** AF detection performance based on RQA features and CTW–RQA features by means of RF.

Features	ACC	PRE	SEN	SPE
RQA	83.50	85.64	80.50	86.50
4 s CTW–RQA (*n* = 6)	81.75	83.74	80.95	82.63
**8 s CTW–RQA (*n* = 3)**	**89.38**	**90.52**	**88.54**	**90.26**
12 s CTW–RQA (*n* = 2)	86.13	86.46	84.91	87.29

**Table 6:** AF detection performance based on time and time-frequency features with and without CTW by means of RF.

Features	ACC	PRE	SEN	SPE
**Without CWT**	**87.63**	**85.89**	**89.20**	**86.13**
4 s CTW (*n* = 6)	79.73	69.21	53.73	77.37
8 s CTW (*n* = 3)	84.63	85.22	50.39	91.73
12 s CTW (*n* = 2)	85.38	83.33	84.83	83.94

**Table 7:** AF detection performance based on energy features with and without CTW by means of RF.

Features	ACC	PRE	SEN	SPE
**Without CWT**	**78.50**	**77.89**	**78.68**	**78.33**
4 s CTW (*n* = 6)	68.15	65.52	36.19	78.95
8 s CTW (*n* = 3)	74.63	69.15	75.24	62.89
12 s CTW (*n* = 2)	76.78	78.54	74.05	77.63

**Table 8:** The calculated results of 13 RQA features based on four feature ranking algorithms.

	Fisher Score	MRMR (×10^−3^)	Chi-Square Test (−lg(p))	Mean (|SHAP Value|) (×10^−2^)
RQA 1	26.7275	15.6968	86.1661	9.16
RQA 2	0.0069	36.1666	76.7835	5.83
RQA 3	0.0614	26.1460	109.6421	9.26
RQA 4	0.0067	7.4060	79.8617	4.03
RQA 5	0.0025	7.5923	126.0331	0.75
RQA 6	0.0117	9.2184	69.9101	7.84
RQA 7	0.0437	5.9155	108.9972	1.38
RQA 8	0.0515	14.0229	63.5000	2.99
RQA 9	0.0445	8.5346	83.1469	1.31
RQA 10	0.0136	14.6556	114.1749	1.69
RQA 11	0.0463	24.4646	102.5003	2.02
RQA 12	0.0282	6.8314	95.5515	3.84
RQA 13	0.0282	16.0161	118.5287	2.12

**Table 9:** AF detection performance based on the fusion features.

Method	ACC	PRE	SEN	SPE
KNN	58.13	84.07	23.06	95.36
NB	81.63	85.52	77.42	86.08
ENS	91.75	95.29	88.35	95.36
**RF**	**95.63**	**95.84**	**95.63**	**95.62**
DT	84.50	85.82	83.74	85.31

**Table 10:** AF detection accuracy based on the ranked features.

Method	Top 13	Top 16	Top 17
Fisher’s coefficient	91.25%	89.63%	90.88%
MRMR	92.88%	93.13%	93.63%
**Chi-square test**	**95.25%**	**95.25%**	**95.38%**
SHAP value	93.75%	94.13%	94.63%

## Appendix A

**Table A1:** AF detection performance based on RQA features.

Method	ACC	PRE	SEN	SPE
KNN	56.38	59.41	40.25	72.50
NB	62.88	66.78	51.25	74.50
ENS	77.88	80.38	73.75	82.00
**RF**	**83.50**	**85.64**	**80.50**	**86.50**
DT	79.63	79.70	79.50	79.75

**Table A2:** AF detection performance based on CTW–RQA features (*n* = 6).

Method	ACC	PRE	SEN	SPE
KNN	56.50	65.52	36.19	78.95
NB	69.38	69.15	75.24	62.89
ENS	75.75	78.54	74.05	77.63
**RF**	**81.75**	**83.74**	**80.95**	**82.63**
DT	72.25	72.92	75.00	69.21

**Table A3:** AF detection performance based on CTW–RQA features (*n* = 3).

Method	ACC	PRE	SEN	SPE
KNN	59.25	65.56	43.17	76.15
NB	56.63	54.21	99.02	12.05
ENS	81.25	80.95	82.93	79.49
**RF**	**89.38**	**90.52**	**88.54**	**90.26**
DT	78.88	78.76	80.49	77.18

**Table A4:** AF detection performance based on CTW–RQA features (*n* = 2).

Method	ACC	PRE	SEN	SPE
KNN	63.13	67.52	47.31	78.24
NB	61.13	55.85	97.70	26.16
ENS	82.13	81.47	82.10	82.15
**RF**	**86.13**	**86.46**	**84.91**	**87.29**
DT	79.75	78.70	80.31	79.22

**Table A5:** AF detection performance based on time and time-frequency features.

Method	ACC	PRE	SEN	SPE
KNN	65.88	69.21	53.73	77.37
NB	71.63	85.22	50.39	91.73
ENS	84.38	83.33	84.83	83.94
**RF**	**87.63**	**85.89**	**89.20**	**86.13**
DT	79.38	76.79	82.52	76.40

**Table A6:** AF detection performance based on energy features.

Method	ACC	PRE	SEN	SPE
KNN	58.38	59.00	50.76	65.76
NB	72.13	68.31	80.96	63.55
ENS	75.50	74.03	77.41	73.65
**RF**	**78.50**	**77.89**	**78.68**	**78.33**
DT	73.63	72.82	74.11	73.15
